# Differences in treatment response and survival between HER2(2+)/FISH-positive and HER2(3+) breast cancer patients after dual-target neoadjuvant therapy: a matched case-control study

**DOI:** 10.3389/fonc.2025.1530793

**Published:** 2025-04-04

**Authors:** Sicheng Zhou, Xuhui Qin, Wei Xing, Zhao Xu, Chunlv Wei, Yining Ren, Zixing Gong

**Affiliations:** ^1^ Department of Thyroid and Breast Surgery, Peking University First Hospital, Beijing, China; ^2^ Department of General Surgery, Zanhuang County Hospital of Traditional Chinese Medicine, Shijiazhuang, China; ^3^ Department of General Surgery, Hebei Provincial Hospital of Chinese Medicine/The First Affiliated Hospital of Hebei University of Chinese Medicine, Shijiazhuang, China

**Keywords:** breast cancer, human epidermal growth factor receptor 2, targeted therapy, neoadjuvant therapy, pathological complete response, prognosis

## Abstract

**Background:**

The efficacy of neoadjuvant therapy (NAT) comprising dual-target drugs has been confirmed among patients with human epidermal growth factor receptor 2 (HER2)-positive breast cancer (BC). Therefore, we explored the differences in responses to NAT and prognosis between patients with HER2(3+) and HER2(2+)/fluorescence *in-situ* hybridization (FISH)-positive BC after TCbHP-based dual-target NAT.

**Methods:**

Data from patients with HER2-positive invasive BC who underwent NAT and radical surgery between January 2019 and December 2022 at the Peking University First Hospital and Cancer Hospital of Chinese Academy of Medical Sciences were retrospectively summarized. Propensity score matching (PSM) was used to reduce confounding effects. Pathological complete response (pCR) and invasive disease-free survival (IDFS) were evaluated to respectively reflect therapeutic response and patients’ survival status.

**Results:**

We selected 132 BC patients (66 pairs) through PSM form a cohort of 308 patients. The pCR rate of patients in the HER2(3+) group was significantly higher than that in the HER2(2+)/FISH-positive group after NAT (*P*<0.001). Univariate and multivariate logistic regression analyses determined that pCR was significantly affected by tumor grade, hormone receptor (HR) status, HER2 status (*P*<0.05). The 3-year IDFS rate of HER2(3+) BC patients was better than that of HER2(2+)/FISH-positive BC patient (*P*=0.083), although the difference was not statistically significant. Furthermore, multivariable Cox regression analysis exhibited that positive lymph node, HER2(3+), and pCR were independent prognostic factors for IDFS.

**Conclusion:**

HER2(2+)/FISH-positive BC patients exhibited worse treatment response and prognosis than HER2(3+) BC patients after dual-target NAT, indicating that HER2 expression level is a crucial factor influencing the therapeutic efficacy and prognosis of BC patients after TCbHP-based dual-target NAT.

## Background

According to the global cancer statistics for 2025, breast cancer (BC) is a leading malignancy in women (1). As a heterogeneous group of diseases, BC can be classified into various subtypes based on the expression levels of estrogen receptor (ER), progesterone receptor (PR), human epidermal growth factor receptor 2 (HER2) and proliferation cell nuclear antigen (Ki67). Among them, the HER2-positive subtypes constitute around 15% of the entire BC population, which have the characteristics of strong aggressiveness, high heterogeneity and poor prognosis (2). Neoadjuvant therapy (NAT) utilizing anti-HER2 drugs has become an important strategy for managing HER-2 positive BC patients because it can significantly improve the patients’ pathologic complete response (pCR), an important indicator reflecting treatment response and patient prognosis (3). Recently, NAT regimens comprising dual-target drugs, such as the combinations of trastuzumab plus pertuzumab (NeoSphere and PEONY) and trastuzumab plus small molecule tyrosine kinase inhibitor (NeoALLTO), have been extensively utilized in HER2-positive BC management due to their superior effects (almost twice the pCR rate) compared with single-target therapies (4–6). Therefore, a new era of employing dual-target NAT for HER-2 positive BC management has dawned. The choice of chemotherapy agents used in combination with targeted agents in dual-target NAT regimens can evidently affect their efficacy. Several prospective clinical trials such as KRISTINE, TRAIN-2, and TRYPHENA showed that, compared with traditional NAT based on anthracyclines, NAT based on the combination of paclitaxel and platinum drugs with anti-HER2 drugs (TCbHP) could achieve higher pCR rate, better long-term survival, and lower cardiac toxicity in HER2-positive BC patients (7–9). Therefore, TCbHP has become the first-line regimen of dual target NAT for HER2-positive BC, as recommended by the National Comprehensive Cancer Network (NCCN) and Chinese Society of Clinical Oncology (CSCO) guidelines (10–12).

At present, HER2 expression status remains a recognized key factor for predicting the efficacy of targeted therapies (13). HER2-positive BC can be further divided into HER2(3+) and HER2(2+)/fluorescence *in-situ* hybridization (FISH)-positive subtypes based on immunohistochemistry (IHC) and FISH results (14). It has been demonstrated that HER2(3+) BC patients could achieve higher pCR rates compared with HER2(2+)/FISH-positive BC patients after targeted therapy. However, due to considerable variations in therapeutic strategies adopted by previous research and the scarcity of related investigations, the impact of HER2 expression status on the efficacy and prognostic differences of TCbHP-based dual-target NAT in BC patients remains largely elusive (15–19). Therefore, we herein explored the differences in prognosis and responses to TCbHP-based dual-target NAT between HER2(3+) and HER2(2+)/FISH-positive BC patients and the corresponding predictive clinicopathological factors.

## Participants and methods

### Participant selection

This is a retrospective case-control study carried out using a prospectively collected breast cancer database from two institutions between January 2015 and December 2022. Patients diagnosed with HER2-positive invasive BC who received NAT and underwent radical surgery between January 2019 and December 2021 at the Peking University First Hospital and Cancer Hospital of Chinese Academy of Medical Sciences were retrospectively reviewed for their eligibility. The inclusion criteria were: (1) age between 18-75 years; (2) dual-target NAT with TCbHP; (3) HER2-positive; and (4) clinical stage I-III. The exclusion criteria were as follows: (1) distant metastasis; (2) other neoadjuvant regimens or less than 6 cycles of NAT; (3) incomplete data; (4) occult BC, bilateral BC, or inflammatory BC; (5) a previous history of other malignancies. Finally, 308 eligible patients were included in this study, all of which provided written informed consent. The present study was approved by the ethics committee of the Peking University First Hospital and Cancer Hospital, Chinese Academy of Medical Sciences (approval number: (2023-479-002).

### Preoperative evaluation

All patients underwent mammography, breast ultrasound, and breast contrast magnetic resonance imaging (MRI) to detect and assess the statuses of the primary tumor and lymph node invasion before NAT. The patients were further evaluated by computed tomography (CT) and nuclear medicine examinations [positron emission tomography-computed tomography (PET-CT) if necessary] to exclude distant metastases or other malignancies. Core needle biopsy was performed for pathological and IHC assays. Demographic and clinicopathologic characteristics of the patients, including age, Eastern Cooperative Oncology Group (ECOG) score, body mass index (BMI), menstrual state, family history of breast or ovarian cancer, clinical T stage, clinical N stage, tumor grade, ER status, PR status, hormone receptor (HR) status, Ki67 expression, HER2 expression status as detected by IHC or FISH, and pCR, were collected and analyzed.

### Diagnosis and treatment

Specimens from each enrolled patient were examined and evaluated individually by two pathologists, and if inconsistent results occurred, a third physician was involved and fully discussed. TNM staging was performed based on the American Joint Committee on Cancer (AJCC) staging system (8th edition) (20). ER, PR, and HER2 status was evaluated by IHC according to the diagnostic criteria of the American Society of Clinical Oncology (ASCO)/College of American Pathologist (CAP) (21). ER/PR status was considered positive when the tumor nuclear staining was ≥1%; while HR-positive and HR-negative respectively mean ER and/or PR-positive and ER and PR double negative. Both HER2(2+)/FISH-positive and HER2(3+) were defined as HER2-positive (14). Patients were divided into HER2(3+) and HER2(2+)/FISH-positive groups according to the degree of HER2 expression. According to 2013 St. Gallen consensus, lesions with ≥20% and <20% cells expressing Ki67 were respectively defined as tumors with high and low Ki67 expression (22). All patients received 6 cycles of TCbHP regimen (docetaxel, carboplatin, trastuzumab and pertuzumab) for NAT, and radical surgery was performed 3 to 4 weeks after NAT.

The Miller-Payne (MP) grading system was used to evaluate the efficacy of NAT. pCR was defined as the absence of invasive carcinoma components in breast lesions (G5) and lymph nodes, but the presence of residual ductal carcinoma *in situ* components is allowed (ypT0/is N0) (23).

### Adjuvant therapy and follow-up

A total of 18 cycles of adjuvant therapy were performed within 4 weeks after surgery. Patients with MP grade 3-5 were recommended to receive pertuzumab and trastuzumab, while those who achieve grade 1-2 were given trastuzumab emtansine (T-DM1) (24). Patients received adjuvant endocrine therapy and/or radiotherapy according to the NCCN guidelines, generally in combination with anti-HER2 therapy (25). All patients were recommended to undergo outpatient follow-up once every 3 months within 2 years post-operation, then once every 6 months from the third to the fifth year post-operation, with December 31, 2023 set as the follow-up deadline. Invasive disease-free survival (IDFS), which was defined as the time from operation to the first occurrence of ipsilateral locoregional invasive BC, contralateral invasive BC, distant recurrence, or death from any cause, was determined to appraise prognosis.

### Statistical analysis

Patients in the HER2(3+) and HER2(2+)/FISH-positive groups were matched in a 1:1 ratio through propensity score-matching using the following variables: age, ECOG score, BMI, family history of breast or ovarian cancer, cT stage, cN stage, tumor grade, ER status, PR status, HR status and Ki67 expression.

Categorical variables are expressed as frequencies and percentages, and continuous variables were represented as means ± standard deviations. Univariate logistic regression models were established to identify factors associated with the occurrence of pCR. Variables with *P*< 0.05 in the univariate analyses were included in a multivariate logistic regression model. All significant univariate variables were also applied in a multivariate Cox regression model, and their independent prognostic value was evaluated. The Kaplan-Meier method was utilized for survival analysis, with the outcomes compared using the log rank test. A *P* value less than 0.05 indicates statistical significance. SPSS 27.0 for Windows (IBM Corp, Armonk, NY, USA) was used for data analysis.

## Results

### Demographic and clinicopathologic characteristics of the participants

**Figure 1:**
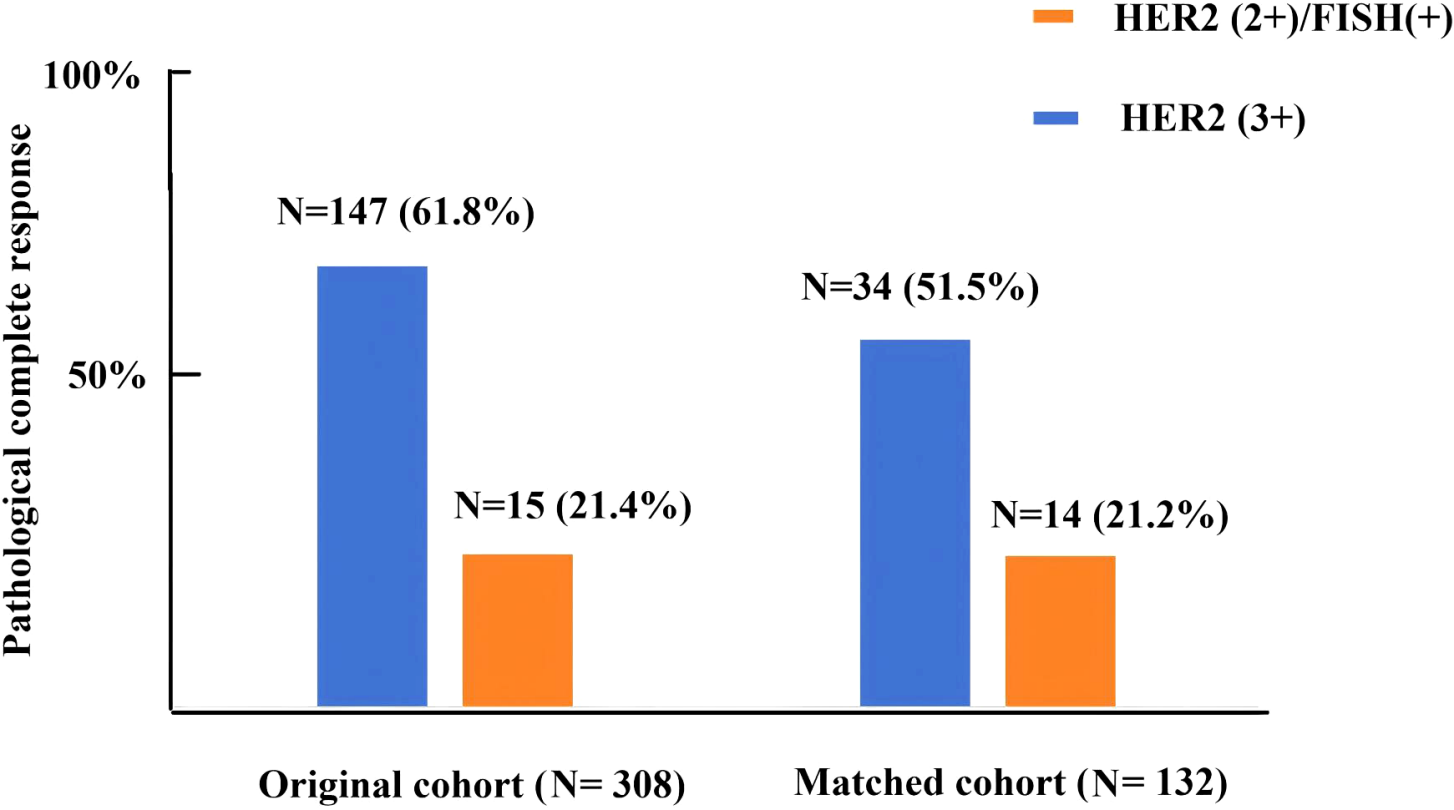
Pathologic complete response rate in HER2(3+) and HER2(2+)/FISH-positive breast cancer patients after receiving dual-target neoadjuvant therapy in original and matched cohorts.

A total of 308 HER2-positive BC patients were recruited in the present study and the basic information of the patients is listed in **Table 1**. All patients were divided into the HER2(3+) (n=238) and HER2(2+)/FISH-positive groups (n=70). Following the process of PSM, 66 matched pairs were eventually selected (**Table 2**). Before PSM, the HER2(2+)/FISH-positive group exhibited significantly higher proportions of ER-positive (81.4% vs. 46.6%, *P*<0.001), PR-positive (68.6% vs. 30.3%, *P*<0.001) and HR-positive (81.4% vs. 48.7%, *P*<0.001) patients than the HER2(3+) group. After NAT, the HER2(3+) group displayed a significantly higher pCR rate (61.8% vs. 21.4%, *P*<0.001) relative to the HER2(2+)/FISH-positive group (**Figure 1**). After PSM, although variations in most of the above mentioned variables became insignificant (*P >*0.05), the HER2(3+) group still had a significantly higher pCR rate (51.5% vs. 21.2%, *P*<0.001) compared with the HER2(2+)/FISH-positive group after NAT (**Figure 1**). In addition, in terms of adjuvant therapy drug used, the proportion of patients using TDM-1 after surgery in the HER2(3+) group was lower than that in the HER2 (2+)/FISH-positive group both in the original (10.5% vs. 22.9%, *P*<0.001) and matched (13.7% vs. 22.7%, *P*=0.176) cohorts, although the difference for the matched cohorts was statistically insignificant.

**Table 1:** Baseline data.

Variables	Total cohort (n = 308)
Age (years, mean±SD)	47.9 ± 9.0
ECOG	
0-1	209 (67.9)
2	99 (22.1)
BMI (kg/m^2^, mean±SD)	21.5 ± 3.0
Menstrual state	
Yes	179 (58.1)
No	129 (41.9)
Family history of breast or ovarian cancer	
Yes	51 (16.6)
No	257 (83.4)
cT stage	
cT1-T2	267 (86.7)
cT3-T4	41 (13.3)
cN stage	
Negative	129 (41.9)
Positive	179 (58.1)
Tumor grade	
I-II	139 (45.1)
III	169 (54.9)
ER status	
ER positive	168 (54.5)
ER negative	140 (45.5)
PR status	
PR positive	120 (39.0)
PR negative	188 (61.0)
HR status	
HR positive	173 (56.2)
HR negative	135 (43.8)
Ki67 expression	
<20%	13 (4.2)
≥20%	295 (95.8)
pCR	
Yes	162 (52.6)
No	148 (47.4)
Adjuvant therapy	
Trastuzumab and pertuzumab	267 (86.7)
Trastuzumab emtansine	41 (13.3)

ECOG, Eastern Cooperative Oncology Group; BMI, body mass index; ER, estrogen receptor; PR, progesterone receptor; HR, hormone receptor; pCR, pathological complete response.

**Table 2:** Baseline demographic and clinicopathologic characteristics of the original and matched cohorts.

Variables	Original cohort (n = 308)			Matched cohort (n = 132)		
	HER2 (3+) N=238	HER2 (2+) /FISH-positive N=70	*P*	HER2 (3+) N=66	HER2 (2+) FISH-positive N=66	*P value*
Age (years, mean±SD)	47.5 ± 9.4	48.5 ± 8.1	0.670	47.7 ± 9.8	48.4 ± 8.2	0.625
ECOG			0.308			0.460
0-1	165 (69.3)	44 (62.9)		46 (69.7)	42 (66.7)	
2	73 (30.7)	26 (37.1)		20 (30.3)	24 (33.3)	
BMI (kg/m^2^, mean±SD)	21.4 ± 2.8	21.9 ± 3.2	0.514	21.6 ± 3.4	21.9 ± 3.4	0.602
Menstrual state			0.523			0.434
Yes	136 (57.1)	43 (61.4)		50 (75.8)	46 (69.7)	
No	102 (42.9)	27 (38.6)		16 (24.2)	20 (30.3)	
Family history of breast or ovarian cancer			0.343			0.436
Yes	42 (17.6)	9 (12.9)		10 (15.2)	7 (10.6)	
No	196 (82.4)	61 (87.1)		56 (84.8)	59 (89.4)	
cT stage			0.501			0.411
cT1-T2	208 (87.4)	59 (84.3)		60 (90.9)	57 (86.4)	
cT3-T4	30 (22.6)	11 (15.7)		6 (9.1)	9 (13.6)	
cN stage			0.851			0.598
Negative	99 (41.6)	30 (42.9)		27 (40.9)	30 (45.5)	
Positive	139 (58.4)	40 (57.1)		39 (59.1)	36 (54.4)	
Tumor grade			0.911			0.861
I-II	107 (45.0)	32 (45.7)		31 (47.0)	30 (45.5)	
III	131 (55.0)	38 (54.3)		35 (53.0)	36 (54.4)	
ER status			<0.001			0.436
ER positive	111 (46.6)	57 (81.4)		59 (89.4)	57 (86.4)	
ER negative	127 (53.4)	13 (18.6)		7 (10.6)	9 (13.6)	
PR status			<0.001			0.573
PR positive	72 (30.3)	48 (68.6)		44 (66.7)	47 (71.2)	
PR negative	166 (69.7)	22 (31.4)		22 (33.3)	19 (28.8)	
HR status			<0.001			0.411
HR positive	116 (48.7)	57 (81.4)		60 (90.9)	57 (86.4)	
HR negative	122 (51.3)	13 (18.6)		6 (9.1)	9 (13.6)	
Ki67 expression			0.296			1.000
<20%	8 (3.4)	5 (7.1)		3 (4.8)	2 (3.0)	
≥20%	230 (96.6)	65 (92.9)		63 (95.2)	64 (97.0)	
pCR			<0.001			<0.001
Yes	147 (61.8)	15 (21.4)		34 (51.5)	14 (21.2)	
No	91 (38.2)	55 (78.6)		32 (48.5)	52 (78.8)	
Adjuvant therapy			0.007			0.176
Trastuzumab and pertuzumab	213 (89.5)	54 (77.1)		57 (86.3)	51 (77.3)	
Trastuzumab emtansine	25 (10.5)	16 (22.9)		9 (13.7)	15 (22.7)	

HER2, human epidermal growth factor receptor 2; FISH, fluorescence in-situ hybridization; ECOG, Eastern Cooperative Oncology Group; BMI, body mass index; ER, estrogen receptor; PR, progesterone receptor; HR, hormone receptor; pCR, pathological complete response.

### Factors associated with pCR

Univariate analyses (**Table 3**) showed that tumor grade (*P*=0.019), HR status (*P*<0.001), and HER2 status (*P*<0.001) were significantly associated with increased pCR possibility, while the associations between pCR occurrence and other factors were not significant (*P*>0.05). All significant univariate variables were applied in a multivariate analysis, which showed that tumor grade [odds ratio (OR), 2.293, 95% confidence interval (CI), 1.340-3.923; *P*=0.002), HR status (OR, 2.878, 95% CI, 1.677-4.940; *P*<0.001), HER2 status (OR, 4.861, 95% CI, 2.051-9.382; *P*<0.001) all exhibited statistically significant associations with pCR occurrence (**Table 4**).

**Table 3:** Univariate analyses to predict pathological complete response after neoadjuvant therapy.

	Univariate analysis		pCR
	OR(95%CI)	*P*	
Age at operation (years)		0.618	
≤35	Reference	Reference	50.0
>35 and ≤55	1.097 (0.539-2.233)	0.799	52.3
>55	1.400 (0.627-3.128)	0.412	58.3
Menstrual state			
Yes	Reference	Reference	52.6
No	1.080 (0.685-1.703)	0.741	54.5
Family history of breast or ovarian cancer			
Yes	Reference	Reference	58.3
No	0.772 (0.452-1.318)	0.344	51.9
cT stage			
cT1-T2	Reference	Reference	54.5
cT3-T4	0.714 (0.364-1.402)	0.328	46.2
cN stage			
Negative	Reference	Reference	58.1
Positive	0.720 (0.455-1.139)	0.161	50.0
Tumor grade			
I-II	Reference	Reference	47.3
III	1.735 (1.097-2.744)	0.019	60.9
HR status			
HR positive	Reference	Reference	39.3
HR negative	3.804 (2.344-6.173)	<0.001	71.1
HER2 status			
2+/ FISH-positive	Reference	Reference	21.4
3+	5.536 (3.440-9.463)	<0.001	61.8
Ki67 expression			
<20%	Reference	Reference	50.0
≥20%	1.143 (0.732-2.758)	0.522	54.9

NAT, neoadjuvant therapy; HER2, human epidermal growth factor receptor 2; FISH, fluorescence in-situ hybridization; HR, hormone receptor; pCR, pathological complete response.

**Table 4:** Multivariate analyses to predict pathological complete response after neoadjuvant therapy.

	Multivariate analysis	
	OR(95%CI)	*P*
Tumor grade		
I-II	Reference	
III	2.293 (1.340-3.923)	0.002
Hormone receptor status		
HR positive	Reference	
HR negative	2.878 (1.677-4.940)	<0.001
HER2 status		
2+/ FISH-positive	Reference	
3+	4.861 (2.051-9.382)	<0.001

NAT, neoadjuvant therapy; HER2, human epidermal growth factor receptor 2; FISH, fluorescence in-situ hybridization; HR, Hormone receptor; pCR, pathological complete response.

### Survival analyses

**Figure 2:**
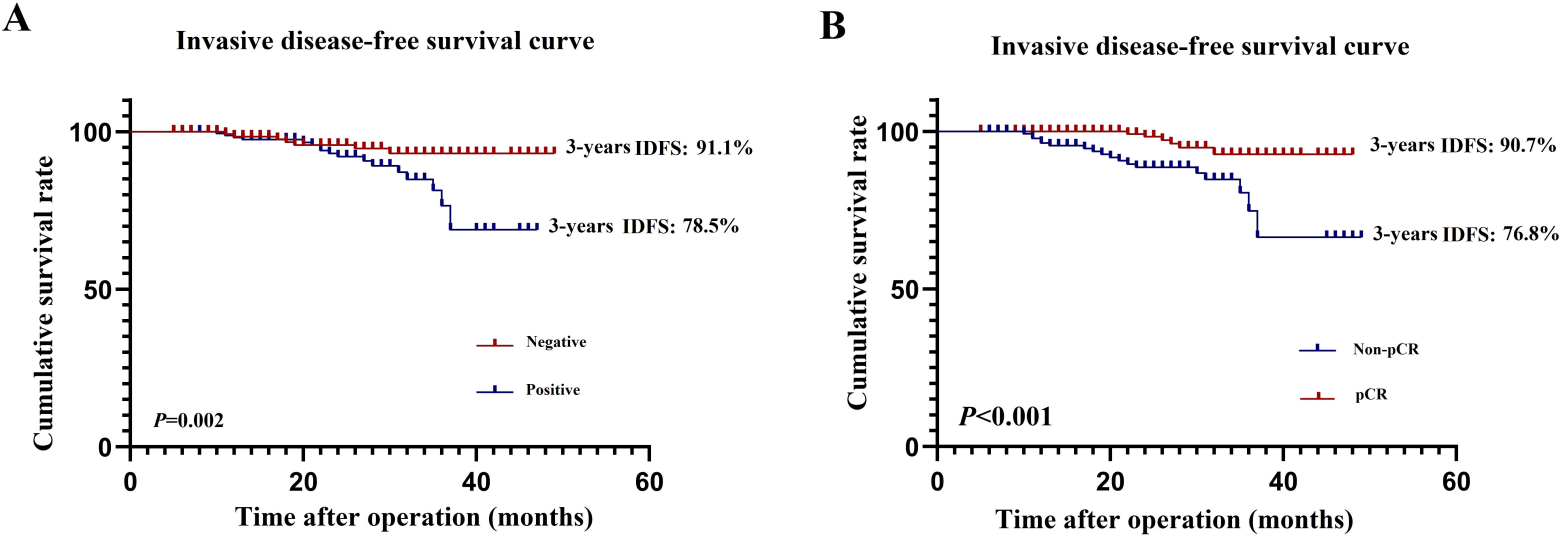
Invasive diseases-free survival in HER2(3+) and HER2(2+)/FISH-positive breast cancer patients after receiving dual-target neoadjuvant therapy in original **(A)** and matched **(B)** cohorts.

The median follow-up periods in the original and matched cohorts were 37 and 40 months, respectively. No patients were lost to follow-up. In the original cohort, patients in the HER2(3+) group had significantly better 3-year IDFS (86.8% vs. 77.2%, *P*=0.016) than those in the HER2 (2+)/FISH-positive group (**Figure 2A**). In the matched cohort, patients in the HER2(3+) group also achieved better 3-year IDFS (90.7% vs. 81.9%, *P*=0.083) than those in the HER2(2+)/FISH-positive group, although the difference was not statistically significant (**Figure 2B**).

**Figure 3:**
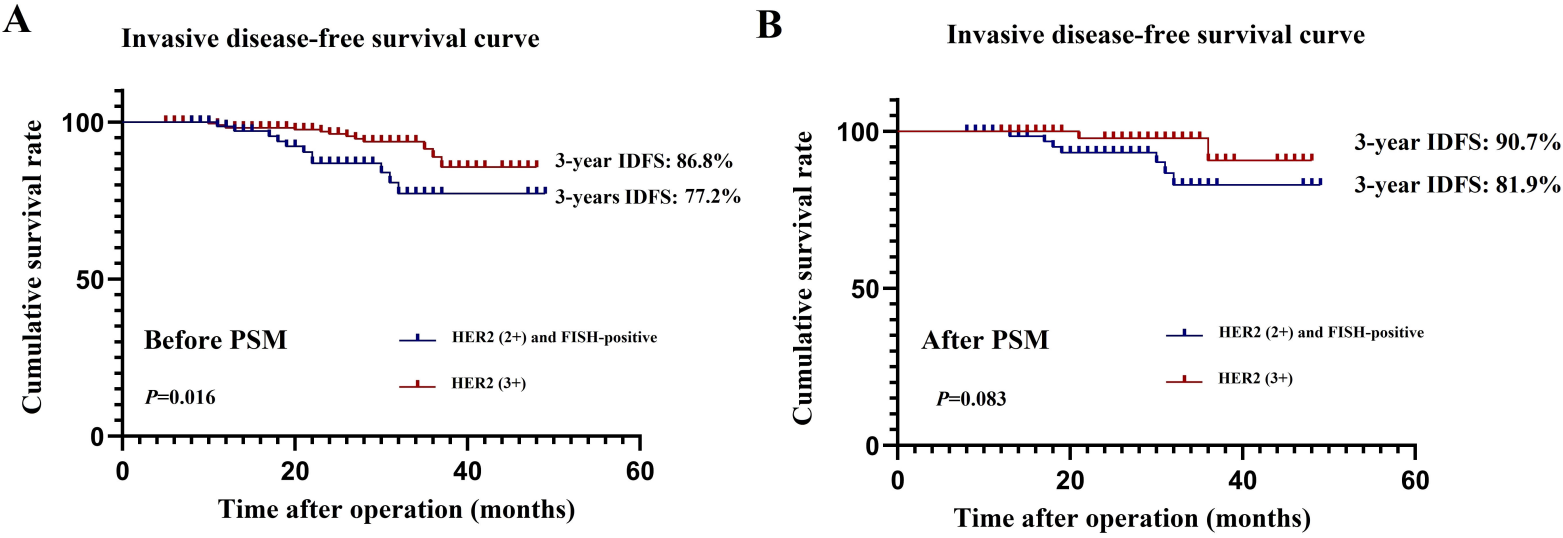
Effect of lymph node status **(A)** and pathological complete response **(B)** on invasive diseases-free survival.

Univariate and multivariate Cox regression analyses were performed on the 308 patients in the original cohort to determine the prognostic factors for HER2-positive BC patients who underwent TCbHP-based dual-target NAT followed by radical surgery (**Table 5**). Univariate regression analyses identified cN stage (*P*=0.002), HER2 status (*P*=0.016), and pCR (*P*<0.001) as factors significantly associated with IDFS. Furthermore, the 3-year IDFS of patients with positive lymph nodes was significantly worse than that of those with negative lymph nodes (91.1% vs. 78.5%, *P*=0.002) (**Figure 3A**). In addition, patients who achieved pCR possessed significantly better 3-year IDFS than those who did not (90.7% vs. 76.8%, *P*<0.001) (**Figure 3B**). In the multivariate Cox regression analysis, three variables emerged as independent prognostic factors, namely positive lymph node [hazard ratio (HR), 2.523, 95% CI, 1.321-10.144; *P*=0.024], HER2(3+) (HR, 0.485, 95% CI, 0.324-0.670; *P*=0.007) and pCR (HR, 0.385, 95% CI, 0.224-0.598; *P*=0.001).

**Table 5:** Univariate and multivariate Cox regression analysis of invasive disease-free survival in original cohort.

Variables	Invasive disease-free survival			
	Univariate analysis		Multivariate analysis	
	HR(95%CI)	*P*	HR(95%CI)	*P*
Age at operation (years)				
≤35	Reference			
>35 and ≤55	0.566 (0.311-1.736)	0.113		
>55	0.626 (0.358-1.905)	0.195		
Menstrual state (yes vs. no)	0.559 (0.220-1.419)	0.221		
Family history of breast or ovarian cancer (yes vs. no)	0.475 (0.142-1.595)	0.228		
cT stage (T3-T4 vs. T1-T2)	4.832 (0.842-10.347)	0.221		
cN stage (positive vs. negative)	5.052(1.892-15.774)	0.002	2.523 (1.321-10.144)	0.024
Tumor grade (III vs. I-II)	2.174 (0.861-5.488)	0.110		
Hormone receptor status (positive vs. negative)	0.770 (0.345-1.721)	0.524		
HER2 status (3+ vs. 2+/FISH-positive)	0.369 (0.165-0.828)	0.016	0.485 (0.324-0.670)	0.007
Ki67 expression ( ≥20% vs. <20%)	1.840 (0.732-3.922)	0.611		
pCR (yes vs. no)	0.308 (0.182-0.564)	<0.001	0.385 (0.224-0.598)	0.001

HER2, human epidermal growth factor receptor 2; FISH, fluorescence in-situ hybridization; HR, Hormone receptor; pCR, pathological complete response.

## Discussion

Randomized controlled trials such as NeoSphere, PEONY and NeoALLTO have confirmed the efficacy of NAT based on trastuzumab in combination with pertuzumab + cytotoxic drugs for patients with HER2-positive BC (4–6). Since 2020, based on evidence from well-designed studies such as KRISTINE, TRAIN-2, and TRYPHENA, both NCCN and CSCO guidelines have prioritized the TCbHP regimen as a preferred option in NAT for HER2-positive BC patients (7–9). In this context, we herein investigated the clinicopathological factors affecting pCR and prognosis of HER2-positive BC patients who underwent TCbHP-based dual-target NAT. Our findings indicate that, after being managed with TCbHP-based dual-target NAT, the pCR rate of HER2(3+) BC patients was significantly higher than that of those with HER2(2+)/FISH-positive lesions. Additionally, tumor grade, HR status, HER2 status and cN stage, HER2 status, pCR were independent factors affecting pCR and IDFS, respectively.

Although both HER2(3+) and HER2(2+)/FISH-positive subtypes belong to HER2-positive BC, the difference in cell surface HER2 protein level between the two subtypes has been suggested to affect their responses to NAT (15–19). For instance, HER2(3+) was found to significantly correlated with a higher pCR compared with the HER2(2+)/FISH-positive subtype (OR=3.71, 95%CI=1.13-12.22, *P*=0.030) (17). Consistent with this observation, Krystel-Whittemore M et al. also noted that the pCR rate of HER2(3+) patients was significantly higher than that of HER2(2+)/FISH-positive patients after NAT (67% vs. 17%, *P*<0.0001) (18). Compared with HER2(3+) tumors, HER2(2+)/FISH-positive lesions were more likely to have lower histologic grade and higher ER and PR positive rates, which per se can result in a lower pCR rate. Therefore, results of the abovementioned studies may be affected by confounding factors. Moreover, the chemotherapy and targeted therapy regimens received by the participants varied between previous studies, with some investigations even adopting multiple treatment regimens, further complicating the impact of HER2 expression level on NAT outcomes. In this study, we only included HER2-positive BC patients who underwent TCbHP-based dual-target NAT and utilized PSM to address baseline confounding factors. Our results demonstrate that patients in the HER2(3+) group had significantly higher pCR rates relative to those in the HER2(2+)/FISH-positive group (51.5% vs. 21.2%, *P*<0.001) after NAT. In addition, multivariate analysis revealed that HER2 status significantly affected the occurrence of pCR (OR, 0.213, 95% CI, 0.152-0.347; *P*<0.001). Our findings were consistent with those of a recent study, which also revealed that HER2(3+) BC patients displayed significantly higher pCR rates compared with HER2(2+)/FISH-positive BC patients after dual-target NAT (41.7% vs. 63.3%, *P*=0.017) (19).

In HER2-positive BC patients managed with NAT, pCR possibility can be affected by clinicopathological factors such as HR status, clinical stage, Ki-67 index, and histological grade (15–19, 26–28). In this study, univariate and multivariate analyses showed that HR status was significantly correlated with pCR (*P*<0.001), that is, HR-negative patients were more likely to display a higher pCR rate than HR-positive ones (71.1% vs. 39.3%, *P*<0.001). Similar to the results of Krystel-Whittemore M et al. (18), the present study also found that the HR-negative rate in the HER2(3+) group was significantly higher than that in the HER2(2+)/FISH-positive group, which may be due to the complex interaction between HR and the HER2 signaling pathway (26). Histological grade evaluation may also be helpful for pCR rate prediction. A previous study demonstrated that the pCR rate of the patients with high histological grade (grade 3) was 70.0%, while that of those with low histological grade (grade 1-2) was only 36.8%. Similarly, this study also found that histological grade was significantly associated with the occurrence of pCR (*P*=0.002). The better treatment efficacy of NAT in high histological grade BC patients may result from the active proliferation of tumor cells and their strong sensitivity to chemotherapy drugs.

As for prognosis, the present study found that the 3-year IDFS of HER2(2+)/FISH-positive patients was worse than that of the propensity score-matched HER2(3+) cohort (90.7% vs. 81.9%, *P*=0.083), although the difference was not statistically significant. Our multivariate analysis also demonstrated that HER2 status was an independent risk factor for poor IDFS (HR, 0.485, 95% CI, 0.324-0.670; *P*=0.007). Furthermore, it has been reported that BC patients’ pCR after management with NAT involving anti-HER2 agents may be a surrogate marker for their prognosis, with those who achieved pCR exhibiting significantly better prognosis (11, 12). We further analyzed if other clinicopathologic factors can affect IDFS in HER2-positive BC patients managed with TCbHP-based dual-target NAT and found that, in addition to pCR, positive lymph node (HR, 2.523, 95% CI, 1.321-10.144; *P*=0.024) was also an independent prognostic factor for IDFS, which was consistent with previous findings reported by the NSABP B-18 and B-27 studies (29, 30). The NSABP B-27 trial showed that, if lymph node was the only parameter analyzed, patients with lymph nodes involvement had significantly worse overall survival rates than those without. In this study, the 3-year IDFS of participants with positive lymph node (78.5%) was significantly worse than that of those with negative lymph node (91.1%, *P*=0.002). Therefore, lymph node involvement should be considered independently in predicting the prognosis of HER2-positive BC patients treated with dual-target NAT.

The present study has several limitations. First, the sample size of the present study is relatively small. Second, the retrospective nature of this study may inevitably introduce selection bias and heterogeneity. Notably, and there were differences in diagnosis and treatment methods adopted between the two participating institutions. Third, emerging biomarkers for HER2-positive BC, such as intrinsic molecular subtypes and tumor-infiltrating lymphocytes, have not been considered in this study. Furthermore, we recommended TDM-1 for patients who achieve MP grade 1-2 after our NAT regimen. However, due to economic reasons and drug-associated side effects, many patients refused to use TDM-1, which may have affected their survival outcomes. Finally, the mean follow-up time of the whole study was less than five years, which is insufficient for accurately evaluating the long-term prognosis.

## Conclusion

In summary, we found that patients with HER2(3+) BC displayed a higher pCR rate than those with HER2(2+)/FISH-positive BC after TCbHP-based dual-target NAT. Additionally, tumor grade, HR status, and HER2 status could determine the efficacy of the NAT regimen. Finally, significantly better prognoses were observed in the BC patients with pCR, HER2(3+), and negative lymph node after this regimen of NAT.

## Data Availability

The raw data supporting the conclusions of this article will be made available by the authors, without undue reservation.

## References

[1] Siegel RL Kratzer TB Giaquinto AN Sung H Jemal A. Cancer statistics, 2025. CA Cancer J Clin. (2025) 75:10–45. doi: 10.3322/caac.21871 39817679 PMC11745215

[2] Slamon DJ Godolphin W Jones LA Holt JA Wong SG Keith DE. Studies of the HER-2/neu proto-oncogene in human breast and ovarian cancer. Science. (1989) 244:707–12. doi: 10.1126/science.2470152 2470152

[3] Gianni L Eiermann W Semiglazov V Manikhas A Lluch A Tjulandin S. Neoadjuvant chemotherapy with trastuzumab followed by adjuvant trastuzumab versus neoadjuvant chemotherapy alone, in patients with HER2-positive locally advanced breast cancer (the NOAH trial): a randomised controlled superiority trial with a parallel HER2-negative cohort. Lancet. (2010) 375:377–84. doi: 10.1016/S0140-6736(09)61964-4 20113825

[4] Baselga J Bradbury I Eidtmann H Di Cosimo S de Azambuja E Aura C. Lapatinib with trastuzumab for HER2-positive early breast cancer (NeoALTTO): a randomised, open-label, multicentre, phase 3 trial. Lancet. (2012) 379:633–40. doi: 10.1016/S0140-6736(11)61847-3 PMC5705192 22257673

[5] Gianni L Pienkowski T Im YH Roman L Tseng LM Liu MC. Efficacy and safety of neoadjuvant pertuzumab and trastuzumab in women with locally advanced, inflammatory, or early HER2-positive breast cancer (NeoSphere): a randomised multicentre, open-label, phase 2 trial. Lancet Oncol. (2012) 13:25–32. doi: 10.1016/S1470-2045(11)70336-9 22153890

[6] Shao Z Pang D Yang H Li W Wang S Cui S. Efficacy, safety, and tolerability of pertuzumab, trastuzumab, and docetaxel for patients with early or locally advanced ERBB2-positive breast cancer in asia: the PEONY phase 3 randomized clinical trial. JAMA Oncol. (2020) 6:e193692. doi: 10.1001/jamaoncol.2019.3692 31647503 PMC6813591

[7] Hurvitz SA Martin M Jung KH Huang CS Harbeck N Valero V. Neoadjuvant trastuzumab emtansine and pertuzumab in human epidermal growth factor receptor 2-positive breast cancer: three-year outcomes from the phase III KRISTINE study. J Clin Oncol. (2019) 37:2206–16. doi: 10.1200/JCO.19.00882 PMC6774816 31157583

[8] van Ramshorst MS van der Voort A van Werkhoven ED Mandjes IA Kemper I Dezentjé VO. Neoadjuvant chemotherapy with or without anthracyclines in the presence of dual HER2 blockade for HER2-positive breast cancer (TRAIN-2): a multicentre, open-label, randomised, phase 3 trial. Lancet Oncol. (2018) 19:1630–40. doi: 10.1016/S1470-2045(18)30570-9 30413379

[9] Schneeweiss A Chia S Hickish T Harvey V Eniu A Hegg R. Pertuzumab plus trastuzumab in combination with standard neoadjuvant anthracycline-containing and anthracycline-free chemotherapy regimens in patients with HER2-positive early breast cancer: a randomized phase II cardiac safety study (TRYPHAENA). Ann Oncol. (2013) 24:2278–84. doi: 10.1093/annonc/mdt182 23704196

[10] Li J Jiang Z. Chinese Society of Clinical Oncology Breast Cancer (CSCO BC) guidelines in 2022: stratification and classification. Cancer Biol Med. (2022) 19:769–73. doi: 10.20892/j.issn.2095-3941.2022.0277 PMC9257320 35765123

[11] Broglio KR Quintana M Foster M Olinger M McGlothlin A Berry SM. Association of pathologic complete response to neoadjuvant therapy in HER2-positive breast cancer with long-term outcomes: A meta-analysis. JAMA Oncol. (2016) 2:751–60. doi: 10.1001/jamaoncol.2015.6113 26914222

[12] Rastogi P Anderson SJ Bear HD Geyer CE Kahlenberg MS Robidoux A. Preoperative chemotherapy: updates of National Surgical Adjuvant Breast and Bowel Project Protocols B-18 and B-27. J Clin Oncol. (2008) 26:778–85. doi: 10.1200/JCO.2007.15.0235 18258986

[13] Piccart-Gebhart MJ Procter M Leyland-Jones B Goldhirsch A Untch M Smith I. Trastuzumab after adjuvant chemotherapy in HER2-positive breast cancer. N Engl J Med. (2005) 353:1659–72. doi: 10.1056/NEJMoa052306 16236737

[14] Tarantino P Hamilton E Tolaney SM Cortes J Morganti S Ferraro E. HER2-low breast cancer: pathological and clinical landscape. J Clin Oncol. (2020) 38:1951–62. doi: 10.1200/JCO.19.02488 32330069

[15] Greenwell K Hussain L Lee D Bramlage M Bills G Mehta A. Complete pathologic response rate to neoadjuvant chemotherapy increases with increasing HER2/CEP17 ratio in HER2 overexpressing breast cancer: analysis of the National Cancer Database (NCDB). Breast Cancer Res Treat. (2020) 181:249–54. doi: 10.1007/s10549-020-05599-1 32277375

[16] Singer CF Tan YY Fitzal F Steger GG Egle D Reiner A. Pathological complete response to neoadjuvant trastuzumab is dependent on HER2/CEP17 ratio in HER2-amplified early breast cancer. Clin Cancer Res. (2017) 23:3676–83. doi: 10.1158/1078-0432.CCR-16-2373 28143867

[17] Spring L Niemierko A Haddad S Yuen M Comander A Reynolds K. Effectiveness and tolerability of neoadjuvant pertuzumab-containing regimens for HER2-positive localized breast cancer. Breast Cancer Res Treat. (2018) 172:733–40. doi: 10.1007/s10549-018-4959-8 PMC6235701 30220055

[18] Krystel-Whittemore M Xu J Brogi E Ventura K Patil S Ross DS. Pathologic complete response rate according to HER2 detection methods in HER2-positive breast cancer treated with neoadjuvant systemic therapy. Breast Cancer Res Treat. (2019) 177:61–6. doi: 10.1007/s10549-019-05295-9 PMC6640097 31144151

[19] Chen W Li FX Lu DL Jiang J Li J. Differences between the efficacy of HER2(2+)/FISH-positive and HER2(3+) in breast cancer during dual-target neoadjuvant therapy. Breast. (2023) 71:69–73. doi: 10.1016/j.breast.2023.07.008 37517155 PMC10400900

[20] Hu J Fung MW Tsang JY Poon IK Chan SK Cheung SY. Improved prognostication for the updated AJCC breast cancer pathological prognostic staging varied in higher-stage groups. Clin Breast Cancer. (2020) 20:253–261.e7. doi: 10.1016/j.clbc.2020.01.011 32205037

[21] Tanioka M Sasaki M Shimomura A Fujishima M Doi M Matsuura K. Pathologic complete response after neoadjuvant chemotherapy in HER2-overexpressing breast cancer according to hormonal receptor status. Breast. (2014) 23:466–72. doi: 10.1016/j.breast.2014.03.008 24742606

[22] Goldhirsch A Winer EP Coates AS Gelber RD Piccart-Gebhart M Thürlimann B. Personalizing the treatment of women with early breast cancer: highlights of the St Gallen International Expert Consensus on the Primary Therapy of Early Breast Cancer 2013. Ann Oncol. (2013) 24:2206–23. doi: 10.1093/annonc/mdt303 PMC3755334 23917950

[23] Guiu S Gauthier M Coudert B Bonnetain F Favier L Ladoire S. Pathological complete response and survival according to the level of HER-2 amplification after trastuzumab-based neoadjuvant therapy for breast cancer. Br J Cancer. (2010) 103:1335–42. doi: 10.1038/sj.bjc.6605939 PMC2990615 20978512

[24] Mamounas EP Untch M Mano MS Huang CS Geyer CE von Minckwitz G. Adjuvant T-DM1 versus trastuzumab in patients with residual invasive disease after neoadjuvant therapy for HER2-positive breast cancer: subgroup analyses from KATHERINE. Ann Oncol. (2021) 32:1005–14. doi: 10.1016/j.annonc.2021.04.011 33932503

[25] Gradishar WJ Moran MS Abraham J Aft R Agnese D Allison KH. Breast cancer, version 3.2022, NCCN clinical practice guidelines in oncology. J Natl Compr Canc Netw. (2022) 20:691–722. doi: 10.6004/jnccn.2022.0030 35714673

[26] Li XB Krishnamurti U Bhattarai S Klimov S Reid MD O'Regan R. Biomarkers predicting pathologic complete response to neoadjuvant chemotherapy in breast cancer. Am J Clin Pathol. (2016) 145:871–8. doi: 10.1093/ajcp/aqw045 27298399

[27] Shokouh TZ Ezatollah A Barand P. Interrelationships between ki67, HER2/neu, p53, ER, and PR status and their associations with tumor grade and lymph node involvement in breast carcinoma subtypes: retrospective-observational analytical study. Med (Baltimore). (2015) 94:e1359. doi: 10.1097/MD.0000000000001359 PMC4616694 26266392

[28] Schneeweiss A Chia S Hickish T Harvey V Eniu A Waldron-Lynch M. Long-term efficacy analysis of the randomised, phase II TRYPHAENA cardiac safety study: Evaluating pertuzumab and trastuzumab plus standard neoadjuvant anthracycline-containing and anthracycline-free chemotherapy regimens in patients with HER2-positive early breast cancer. Eur J Cancer. (2018) 89:27–35. doi: 10.1016/j.ejca.2017.10.021 29223479

[29] Bear HD Anderson S Smith RE Geyer CE Jr Mamounas EP Fisher B. Sequential preoperative or postoperative docetaxel added to preoperative doxorubicin plus cyclophosphamide for operable breast cancer:National Surgical Adjuvant Breast and Bowel Project Protocol B-27. J Clin Oncol. (2006) 24:2019–27. doi: 10.1200/JCO.2005.04.1665 16606972

[30] Ross JS Slodkowska EA Symmans WF Pusztai L Ravdin PM Hortobagyi GN. The HER-2 receptor and breast cancer: ten years of targeted anti-HER-2 therapy and personalized medicine. Oncologist. (2009) 14:320–68. doi: 10.1634/theoncologist.2008-0230 19346299

